# Crystal structure of rubidium methyl­diazo­tate

**DOI:** 10.1107/S2056989016020429

**Published:** 2017-01-13

**Authors:** Tobias Grassl, Nikolaus Korber

**Affiliations:** a Institut für Anorganische Chemie, Universität Regensburg, Universitätsstrasse 31, 93053 Regensburg, Germany

**Keywords:** crystal structure, reductive ammonolysis, streptozocin, rubidium cation, methyl­diazo­tate anion

## Abstract

The title compound, Rb^+^·H_3_CN_2_O^−^, has been crystallized in liquid ammonia as a reaction product of the reductive ammonolysis of the natural compound streptozocin. Elemental rubidium was used as reduction agent as it is soluble in liquid ammonia, forming a blue solution. Reductive bond cleavage in biogenic materials under kinetically controlled conditions offers a new approach to gain access to sustainably produced raw materials. The anion is nearly planar [dihedral angle O—N—N—C = −0.4 (2)°]. The Rb^+^ cation has a coordination number of seven, and coordinates to five anions. One anion is bound *via* both its N atoms, one by both O and N, two anions are bound by only their O atoms, and the last is bound *via* the N atom adjacent to the methyl group. The diazo­tate anions are bridged by cations and do not exhibit any direct contacts with each other. The cations form corrugated layers that propagate in the (-101) plane.

## Abstract 2

The title compound has been crystallized in liquid ammonia as a reaction product of the reductive ammonolysis of the natural compound streptozocin. Elemental rubidium was used as reduction agent as it is soluble in liquid ammonia, forming a blue solution. Reductive bond cleavage in biogenic materials under kinetically controlled conditions offers a new approach to gain access to sustainably produced raw materials.

## Chemical context   

The crystal structure of the title compound was determined in the course of investigations regarding the reactivity of carbohydrates towards alkali metals and NH_3_ in solutions where liquid ammonia itself is used as solvent. The starting material, streptozocin, was commercially available and used as shipped.
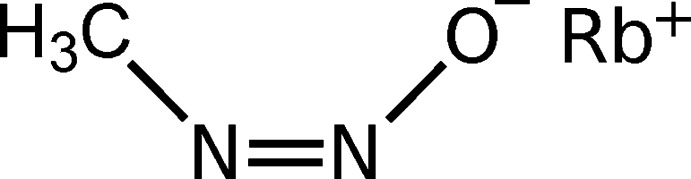


## Structural commentary   

The methyl­diazo­tate anion is found to exist in the *cis* configuration, which is in correspondence with the equivalent potassium species (Müller *et al.*, 1963 ▸; Huber *et al.*, 1965 ▸). The structure of the diazo­tate anion has been further discussed by Suhr (1963 ▸) and by Kübler & Lüttke (1963 ▸).

**Figure 1:**
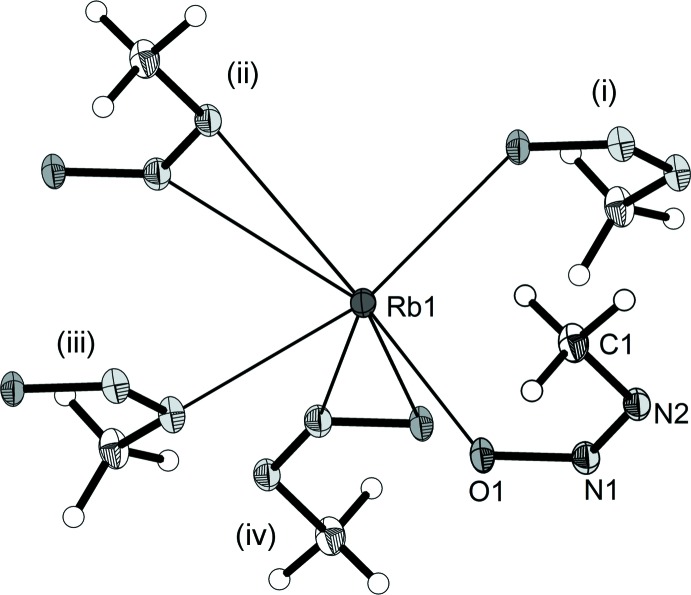
The coordination environment of the Rb^+^ cation. Displacement ellipsoids are drawn at the 50% probability level. [Symmetry codes: (i) −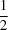 + *x*, 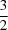 − *y*, 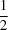 + *z*; (ii) −1 + *x*, *y*, *z*; (iii) −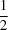 + *x*, 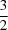 − *y*, 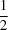 + *z*; (iv) 1 − *x*, 1 − *y*, 1 − *z*.]

The title compound does not contain any solvent mol­ecules, which is unusual for ionic species crystallized from liquid ammonia. The anion is nearly planar, having an O1—N1—N2—C1 torsion angle of −0.4 (2)°. Five direct anion–cation contacts can be observed, with maximum bond lengths of *d*(Rb—O) = 2.9871 (12) Å and *d*(Rb—N) = 3.1656 (15) Å. The rubidium cation has a coordination number of seven, in which five anions can be observed in its direct environment (Fig. 1 ▸). The coordination to the cation is both side-on and terminal: one anion is bound *via* both its N atoms, one by both O and N, two anions are bound only *via* O, and the remaining anion is bound *via* the N atom adjacent to the methyl group.

## Supra­molecular features   

**Figure 2:**
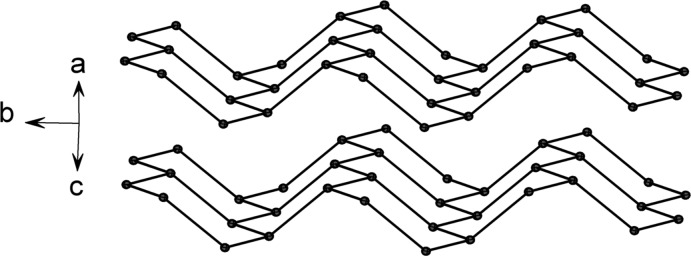
The extended arrangement formed by the cations in the crystal structure. Displacement ellipsoids are drawn at the 50% probability level.

The diazo­tate anions are bridged by cations and do not exhibit any direct contacts to each other. The cations are found to form a corrugated-layer like arrangement within the structure, propagating in the (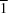
01) plane (Fig. 2 ▸). Although the oxygen atom can act as a hydrogen-bridge acceptor, no such inter­actions can be found in the structure as the C—H bonds are not sufficiently polarized. As the compound is of an ionic nature, electrostatic inter­actions are the dominant driving force towards the arrangement of the ionic species. An aggregation of methyl groups is therefore not observed.

## Computational analysis   

**Figure 3:**
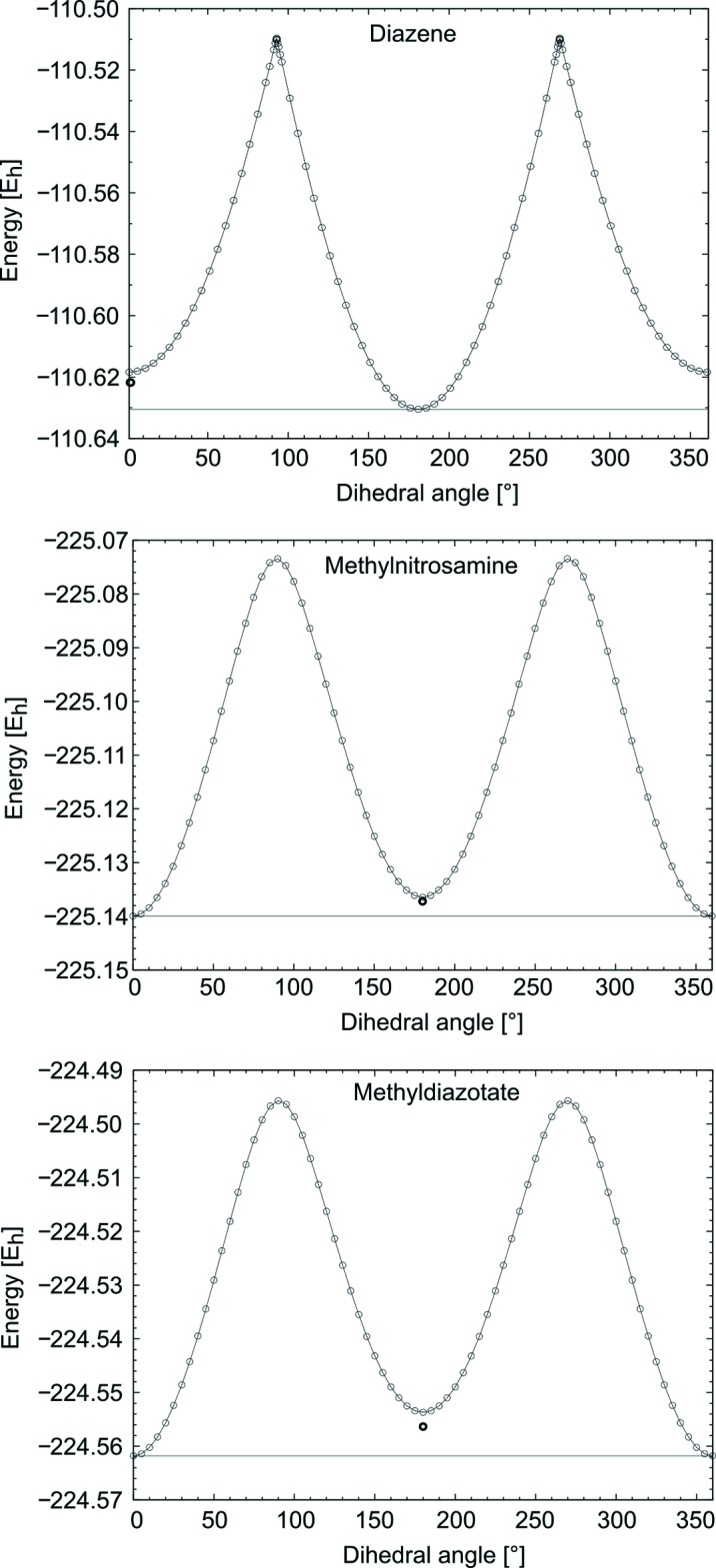
Rotational potentials of diazene, methyl­nitro­samine and methyl­diazo­tate. The energetic minima were geometrically optimized and are drawn as thick circles.

To get a more detailed understanding of the bonding situation in the anion, quantum chemical calculations were carried out at the DFT level (B3LYP functional) using def2-TZVP basis sets. To embed the results in a meaningful frame of reference, diazene and methyl­nitro­samine were used for comparison (Fig. 3 ▸). It was found that the methyl­diazo­tate anion tends to have properties most similar to methyl­nitro­samine. This indicates a high ability to delocalize its *sp* ^2^ electrons.

By analyzing the rotational potential, the energy barrier of the transition between the *cis* and *trans* form was determined to be 173.57 kJ mol^−1^. The energetic difference between the two forms is 14.30 kJ mol^−1^, wherein the *cis* form is energetically preferred. For comparison, the rotational barriers of diazene and methyl­nitro­samine are calculated to be 317.44 kJ mol^−1^ and 174.58 kJ mol^−1^, respectively. The various computational methods employed have been described by Neese (2012 ▸), Weigend & Ahlrichs (2005 ▸), Schäfer *et al.* (1992 ▸, 1994 ▸), Eichkorn *et al.* (1997 ▸), Weigend *et al.* (2003 ▸), Metz *et al.* (2000 ▸), Dirac (1929 ▸), Slater (1951 ▸), Vosko *et al.* (1980 ▸), Becke (1988 ▸, 1993 ▸), Lee *et al.* (1988 ▸).

## Synthesis and crystallization   

250 mg (0.94 mmol) of streptozocin and 322 mg (3.8 mmol) of rubidium were placed under an argon atmosphere in a reaction vessel and 20 ml of dry liquid ammonia was condensed. The mixture was stored at 237 K for two weeks to ensure that all substances were completely dissolved. The flask was then stored at 161 K for several months. After that period, clear colorless crystals of the title compound could be found at the bottom of the flask.

## Refinement   

Crystal data, data collection and structure refinement details are summarized in Table 1 ▸. All hydrogen atoms could be located in a difference map and were refined freely.

## Supplementary Material

Crystal structure: contains datablock(s) I. DOI: 10.1107/S2056989016020429/pk2593sup1.cif

Structure factors: contains datablock(s) I. DOI: 10.1107/S2056989016020429/pk2593Isup2.hkl

CCDC reference: 1524271

Additional supporting information:  crystallographic information; 3D view; checkCIF report

## Figures and Tables

**Table 1:** Experimental details

Crystal data	
Chemical formula	Rb^+^·CH_3_N_2_O^−^
*M*_r_	144.52
Crystal system, space group	Monoclinic, *P*2_1_/*n*
Temperature (K)	123
*a*, *b*, *c* (Å)	6.8658 (1), 8.7614 (1), 7.2447 (1)
β (°)	114.219 (2)
*V* (Å^3^)	397.44 (1)
*Z*	4
Radiation type	Mo *K*α
μ (mm^−1^)	12.26
Crystal size (mm)	0.29 × 0.17 × 0.15

Data collection	
Diffractometer	Agilent SuperNova Dual Source diffractometer with an Eos detector
Absorption correction	Analytical [*CrysAlis PRO* (Agilent, 2012 ▸) based on expressions derived by Clark & Reid (1995 ▸)]
*T*_min_, *T*_max_	0.267, 0.267
No. of measured, independent and observed [*I* > 2σ(*I*)] reflections	13443, 1210, 1068
*R*_int_	0.051
(sin θ/λ)_max_ (Å^−1^)	0.714

Refinement	
*R*[*F* ^2^ > 2σ(*F* ^2^)], *wR*(*F* ^2^), *S*	0.017, 0.042, 1.04
No. of reflections	1210
No. of parameters	58
H-atom treatment	All H-atom parameters refined
Δρ_max_, Δρ_min_ (e Å^−3^)	0.56, −0.86

Computer programs: *CrysAlis PRO* (Agilent, 2012 ▸), *olex2.solve* (Bourhis *et al.*, 2015 ▸), *SHELXL2014* (Sheldrick, 2015 ▸), *DIAMOND* (Brandenburg & Putz, 2012 ▸) and *OLEX2* (Dolomanov *et al.*, 2009 ▸).

## supplementary crystallographic information

### Crystal data

**Table:**

Rb^+^·CH_3_N_2_O^−^	*F*(000) = 272
*M**_r_* = 144.52	*D*_x_ = 2.415 Mg m^−^^3^
Monoclinic, *P*2_1_/*n*	Mo *K*α radiation, λ = 0.71073 Å
*a* = 6.8658 (1) Å	Cell parameters from 8481 reflections
*b* = 8.7614 (1) Å	θ = 3.1–32.1°
*c* = 7.2447 (1) Å	µ = 12.26 mm^−^^1^
β = 114.219 (2)°	*T* = 123 K
*V* = 397.44 (1) Å^3^	Block, colourless
*Z* = 4	0.29 × 0.17 × 0.15 mm

### Data collection

**Table:**

Agilent SuperNova Dual Source diffractometer with an Eos detector	1210 independent reflections
Radiation source: SuperNova (Mo) X-ray Source	1068 reflections with *I* > 2σ(*I*)
Mirror monochromator	*R*_int_ = 0.051
Detector resolution: 15.9702 pixels mm^-1^	θ_max_ = 30.5°, θ_min_ = 3.4°
phi and ω scans	*h* = −9→9
Absorption correction: analytical [CrysAlis PRO (Agilent, 2012) based on expressions derived by Clark & Reid (1995)]	*k* = −12→12
*T*_min_ = 0.267, *T*_max_ = 0.267	*l* = −10→10
13443 measured reflections	

### Refinement

**Table:**

Refinement on *F*^2^	0 restraints
Least-squares matrix: full	Hydrogen site location: difference Fourier map
*R*[*F*^2^ > 2σ(*F*^2^)] = 0.017	All H-atom parameters refined
*wR*(*F*^2^) = 0.042	*w* = 1/[σ^2^(*F*_o_^2^) + (0.0256*P*)^2^] where *P* = (*F*_o_^2^ + 2*F*_c_^2^)/3
*S* = 1.04	(Δ/σ)_max_ = 0.001
1210 reflections	Δρ_max_ = 0.56 e Å^−^^3^
58 parameters	Δρ_min_ = −0.86 e Å^−^^3^

### Special details

**Table:**

Geometry. All esds (except the esd in the dihedral angle between two l.s. planes) are estimated using the full covariance matrix. The cell esds are taken into account individually in the estimation of esds in distances, angles and torsion angles; correlations between esds in cell parameters are only used when they are defined by crystal symmetry. An approximate (isotropic) treatment of cell esds is used for estimating esds involving l.s. planes.

### Fractional atomic coordinates and isotropic or equivalent isotropic displacement parameters (Å^2^)

**Table:**

	*x*	*y*	*z*	*U*_iso_*/*U*_eq_	
Rb1	0.24027 (2)	0.64932 (2)	0.44893 (2)	0.01283 (6)	
O1	0.6929 (2)	0.68315 (14)	0.5959 (2)	0.0159 (2)	
N1	0.7933 (2)	0.67488 (17)	0.4770 (2)	0.0163 (3)	
N2	0.8203 (2)	0.79640 (17)	0.3938 (2)	0.0159 (3)	
C1	0.7331 (3)	0.9368 (2)	0.4396 (3)	0.0194 (3)	
H2	0.583 (3)	0.928 (2)	0.408 (4)	0.027 (6)*	
H3	0.753 (3)	1.018 (2)	0.352 (3)	0.016 (5)*	
H1	0.812 (4)	0.967 (3)	0.587 (5)	0.049 (9)*	

### Atomic displacement parameters (Å^2^)

**Table:**

	*U*^11^	*U*^22^	*U*^33^	*U*^12^	*U*^13^	*U*^23^
Rb1	0.01432 (9)	0.01265 (9)	0.01354 (9)	0.00103 (6)	0.00777 (6)	0.00118 (6)
O1	0.0194 (6)	0.0169 (6)	0.0160 (6)	−0.0005 (4)	0.0120 (5)	0.0004 (5)
N1	0.0190 (7)	0.0167 (8)	0.0158 (7)	0.0019 (5)	0.0098 (6)	0.0009 (6)
N2	0.0204 (7)	0.0145 (6)	0.0163 (7)	0.0029 (6)	0.0112 (6)	0.0019 (6)
C1	0.0294 (9)	0.0126 (8)	0.0223 (9)	0.0019 (7)	0.0167 (8)	0.0002 (7)

### Geometric parameters (Å, º)

**Table:**

Rb1—Rb1^i^	4.2210 (2)	N2—Rb1^v^	3.0313 (14)
Rb1—Rb1^ii^	4.2365 (3)	N2—Rb1^vi^	3.0761 (15)
Rb1—Rb1^iii^	5.2757 (2)	N2—C1	1.465 (2)
Rb1—C1^iv^	3.7471 (18)	C1—Rb1^i^	3.7471 (18)
O1—Rb1^i^	2.8496 (12)	C1—Rb1^vi^	3.6538 (18)
O1—Rb1^ii^	2.9871 (12)	C1—Rb1^vii^	3.7031 (19)
O1—N1	1.3074 (19)	C1—H2	0.97 (2)
N1—Rb1^v^	3.1656 (15)	C1—H3	1.00 (2)
N1—Rb1^ii^	2.9173 (15)	C1—H1	1.02 (3)
N1—N2	1.274 (2)		
			
Rb1^ii^—Rb1—Rb1^iii^	113.582 (6)	Rb1^vii^—C1—Rb1^i^	90.16 (4)
Rb1^i^—Rb1—Rb1^iii^	85.861 (5)	Rb1^vi^—C1—Rb1^i^	156.42 (6)
Rb1^i^—Rb1—Rb1^ii^	77.186 (4)	Rb1^vi^—C1—Rb1^vii^	113.28 (5)
C1^iv^—Rb1—Rb1^ii^	73.60 (3)	Rb1^vii^—C1—H2	94.8 (13)
C1^iv^—Rb1—Rb1^iii^	67.80 (3)	Rb1^i^—C1—H2	78.4 (15)
C1^iv^—Rb1—Rb1^i^	127.36 (3)	Rb1^vi^—C1—H2	101.1 (15)
Rb1^i^—O1—Rb1^ii^	129.33 (5)	Rb1^vi^—C1—H3	58.0 (12)
N1—O1—Rb1^ii^	74.24 (8)	Rb1^vii^—C1—H3	55.4 (12)
N1—O1—Rb1^i^	136.30 (10)	Rb1^i^—C1—H3	144.9 (12)
Rb1^ii^—N1—Rb1^v^	95.44 (4)	Rb1^vii^—C1—H1	63.8 (16)
O1—N1—Rb1^v^	146.43 (12)	Rb1^i^—C1—H1	39.6 (15)
O1—N1—Rb1^ii^	80.21 (8)	Rb1^vi^—C1—H1	149.8 (14)
N2—N1—Rb1^v^	72.28 (9)	N2—C1—Rb1^vii^	152.09 (11)
N2—N1—Rb1^ii^	159.82 (11)	N2—C1—Rb1^i^	102.26 (10)
N2—N1—O1	118.93 (13)	N2—C1—Rb1^vi^	55.69 (8)
Rb1^v^—N2—Rb1^vi^	87.43 (4)	N2—C1—H2	112.1 (13)
N1—N2—Rb1^v^	84.13 (10)	N2—C1—H3	106.3 (11)
N1—N2—Rb1^vi^	128.81 (11)	N2—C1—H1	111.5 (16)
N1—N2—C1	116.27 (13)	H2—C1—H3	108.5 (17)
C1—N2—Rb1^v^	141.49 (11)	H2—C1—H1	109 (2)
C1—N2—Rb1^vi^	101.15 (10)	H3—C1—H1	109.3 (19)

Symmetry codes: (i) *x*+1/2, −*y*+3/2, *z*+1/2; (ii) −*x*+1, −*y*+1, −*z*+1; (iii) −*x*+1/2, *y*+1/2, −*z*+1/2; (iv) *x*−1/2, −*y*+3/2, *z*−1/2; (v) *x*+1, *y*, *z*; (vi) *x*+1/2, −*y*+3/2, *z*−1/2; (vii) −*x*+1, −*y*+2, −*z*+1.
